# Sexual Assault in the Mahdia Region: Medico Legal Aspects

**DOI:** 10.1192/j.eurpsy.2022.720

**Published:** 2022-09-01

**Authors:** S. Brahim, M.A. Mesrati, M. Henia, M. Kacem, L. Zarrouk

**Affiliations:** 1 University Hospital of Mahdia, Psychiatry, chebba, Tunisia; 2 University Hospital of Mahdia, Tunisia., Legal Medecine, mahdia, Tunisia; 3 University Hospital of Mahdia, Tunisia., Psychiatry, mahdia, Tunisia

**Keywords:** SEXUAL ASSAULT, Epidemiology, MEDICO LEGAL ASPECTS

## Abstract

**Introduction:**

Despite recent legislative changes through the enactment of 2017-58 law on the elimination of violence against women and children, sexual violence remains fairly frequent and is often underestimated.

**Objectives:**

to describe the epidemiological peculiarities of victims of sexual assault in the Mahdia region and to discuss their medico legal implications.

**Methods:**

this is retrospective study of 110 cases of victims of sexual assault examined at the legal medicine department of the TAHER SFAR University Hospital in Mahdia. This work was carried out over the period from January 2016 to August 2018.

**Results:**

the majority of victims were female (80%) and the main vulnerability factor was an age under 15 (26%). The perpetrator was generally unique (74%). Sexual assault by penetration was mostly reported (51% of cases), and was almost exclusively penile (98,2 of cases). The gynecological examination revealed a torn hymn in 43 victims, a compliant hymen without traumatic lesions in 7 victims (8%) and recent vulvar traumatic lesions without hymenal crossing in 5 victims (5,6%). Recent anal penetration was diagnosed in 6 male victims (6,8%). Among female victims, recent anal penetration was diagnosed in 5 victims (22,7%). One in four victims reported a market psychological impact with female predominance in 85% of cases. Complications of the most reported sexual assaults were pregnancy in 7% of cases. In total, only 57,3% of the certificates issued made it possible to conclude that the injuries.

**Conclusions:**

The care of victims of sexual assault requires a multi- disciplinary approach; medical, psychiatric, social and legal.

**Disclosure:**

No significant relationships.

